# S-layer architecture and glycosylation and their role in shaping virus–host specificity in prokaryotes

**DOI:** 10.3389/fmicb.2026.1882749

**Published:** 2026-07-22

**Authors:** Sabine Schwarzer, Úlfur Á. Atlason, Tessa E. F. Quax

**Affiliations:** Biology of Archaea and Viruses, Groningen Biomolecular Sciences and Biotechnology Institute, University of Groningen, Groningen, Netherlands

**Keywords:** archaeal virus, bacteriophage, glycosylation, S-layer, virus-host interaction

## Abstract

The crystalline surface layer (S-layer) of prokaryotes constitutes a critical macromolecular interface governing virus–host interactions. In this review, we synthesize recent findings regarding the role of S-layer glycoproteins as viral receptors, examine the structural and functional diversity of these systems across prokaryotic lineages, and analyze the molecular strategies that determine virus–host specificity. By integrating perspectives from structural biology, microbiology, and evolutionary studies, we seek to elucidate the fundamental principles and emerging themes that characterize virus–host interactions at the cell surface mediated by the S-layer.

## Introduction

Surface layers (S-layers) are structures made up of (glyco)proteins that form crystalline lattices covering the surfaces of many prokaryotes, including most archaea and numerous bacteria (Sleytr et al., 2014; Albers and Meyer, 2011; Fagan and Fairweather, 2014). These layers consists of one or two protein subunits that self-assemble into a highly ordered two-dimensional array, typically 2–25 nm thick, creating a continuous protective monolayer that encloses the entire cell (Sleytr et al., 2014; Pum et al., 2013; Rodrigues-Oliveira et al., 2017). The regular symmetrical arrangement of subunits, commonly exhibiting oblique, square, or hexagonal symmetry, creates pores of 2–8 nm in diameter, facilitating selective solute exchange while providing resilience against environmental stress, maintaining cellular integrity, and mediating interactions with the external world (Sleytr et al., 2014; Fagan and Fairweather, 2014; Pum et al., 2013; von Kügelgen et al., 2020; de la Fuente-Núñez et al., 2012; Isbilir et al., 2025). In archaea S-layers frequently serve as the sole component of the cell wall; however, in bacteria and some archaea, they are found alongside or outside other cell wall constituents such as peptidoglycan or pseudomurein (Fagan and Fairweather, 2014; Sivabalasarma et al., 2025; Medvedeva et al., 2024). S-layers are typically anchored to the membrane either through their transmembrane domain or by means of a lipid anchor (Sleytr et al., 2014; Fagan and Fairweather, 2014).

S-layers are particularly prevalent in extremophilic organisms, including thermophilic archaea such as *Pyrococcus furiosus*, *Igniococcus* spp., and *Sulfolobus* spp., as well as bacteria such as *Deinococcus radiodurans, Thermus thermophilus*, and several *Bacillus* spp. In these organisms, S-layers contribute critically to cell envelope stability and functionality under extreme physicochemical conditions (Sleytr et al., 2014; Isbilir et al., 2025; Dyall-Smith, 2008; Schäffer et al., 2002; Zhang et al., 2019; Fernandez-Herrero et al., 1995; Gambelli et al., 2024; Farci et al., 2022; Akca et al., 2002; Claus et al., 2002; Mader et al., 1999; Egelseer et al., 2001). Beyond structural roles, S-layer proteins (SLPs) may contain effector domains, particularly in gram-positive bacteria, that mediate adhesion to host tissues, biofilm formation, or interference with host immune responses (Ravi et al., 2021; Assandri et al., 2023; Gerbino et al., 2015). In pathogenic bacteria, S-layers can therefore act as virulence factors and are increasingly explored as potential therapeutic targets (Assandri et al., 2023; Grill-Walcher and Schäffer, 2025; Dicks and Vermeulen, 2024). Their widespread presence among phylogenetically ancient thermophiles suggests that S-layers were among the earliest cell wall forms, selected for their stability and adaptability in early-Earth environments (Sleytr et al., 2014; Kish et al., 2016; Claus et al., 2005).

S-layers exhibit extensive genetic and structural diversity (Isbilir et al., 2025; Sivabalasarma et al., 2025; Johnston et al., 2024). Many prokaryotes encode multiple SLP genes, and rapid alteration of surface composition in response to environmental pressures is enabled by phase variation (differential expression of SLP under different conditions) or gene-switching mechanisms (Fagan and Fairweather, 2014; Van Der Woude and Bäumler, 2004). This diversity is further expanded by post-translational modifications, most notably glycosylation, which can significantly alter S-layer properties and interactions (Ristl et al., 2010; Schulze et al., 2021; Kandiba and Eichler, 2014; Schuster and Sleytr, 2015). Glycosylation of SLPs is a widespread and often essential modification in both bacteria and archaea (Ristl et al., 2010; Kandiba and Eichler, 2014; Abu-Qarn et al., 2008; Eichler and Adams, 2005; Pohlschroder et al., 2018). This process is typically mediated by dedicated gene clusters encoding enzymes involved in glycan biosynthesis, transport, and covalent attachment to specific residues on SLPs. The resulting glycans can exhibit substantial structural diversity, influencing protein stability, surface charge, and interactions with external factors (Novotny et al., 2004; Nothaft and Szymanski, 2013; Ristl et al., 2010; Jarrell et al., 2014).

In prokaryotes, S-layers function not only as protective cell barriers but also as primary binding sites for bacterial viruses (bacteriophages) and archaeal viruses (Sivabalasarma et al., 2025; Leprince and Mahillon, 2023; Zink et al., 2019; Bertozzi Silva et al., 2016). Bacteriophages and archaeal viruses typically undergo a life cycle comprising attachment, genome entry, replication, assembly, and release of progeny viral particles (Bertozzi Silva et al., 2016; Poranen et al., 2002; Nobrega et al., 2018; Almeida and Abedon, 2023; Dennehy and Abedon, 2021; Leprince et al., 2026). Successful infection depends on the recognition of specific receptors, which are typically exposed on the cell surface. Some examples include polysaccharides, cell-wall teichoic acids, outer-membrane proteins, cell appendages such as pili, flagella, or archaella, and cell-wall components such as S-layers (Leprince and Mahillon, 2023; Bertozzi Silva et al., 2016; Nobrega et al., 2018; Quemin and Quax, 2015; Tittes et al., 2021). Virus adsorption to the host cell may be reversible, permitting detachment under unfavorable conditions, or irreversible, which commits the virus to infection as conformational changes induce genome delivery (Almeida and Abedon, 2023; Dennehy and Abedon, 2021; Letarov and Kulikov, 2017).

Although the molecular details of the initial binding steps remain incompletely understood, recent advances have begun to elucidate the structural and mechanistic basis by which bacteriophages and archaeal viruses recognize and engage S-layer components. This review synthesizes recent advances that shed light on the molecular mechanisms governing the initial attachment of viruses to S-layer components.

## Examples of S-layer functioning as a viral receptor amongst archaea

The first direct evidence of viral binding to S-layer in archaea was observed with Sulfolobus Spindle-shaped virus 1 (SSV1), a chronic virus of the *Fuselloviridae* family (Contursi et al., 2014). Experimental inhibition of S-layer expression of its thermoacidophilic host, *Sulfolobus solfataricus* resulted in reduced infection. Further support for SSV1 binding to the S-layer was provided by the observation that the viral tail structure is complementary in size and shape to pores of the host S-layer (Zink et al., 2019; Stedman et al., 2015).

Among viruses that infect methanogens, the Methanosarcina Spherical Virus (MetSV) is believed to bind to the S-layer of its host, *Methanosarcina mazei* Gö1. The virus attaches over the entire host cell surface (Gehlert et al., 2022). In response to predation by MetSV, host cells form aggregates, a process that involves the formation of a methanochondroitin sheath (Kreisl and Kandler, 1986; Weidenbach et al., 2017). Compared with other S-layer-binding viruses, MetSV demonstrates a broader host range, infecting additional non-aggregating *Methanosarcina* strains (Weidenbach et al., 2017).

Attachment to the S-layer is common among viruses of Haloarchaea, occurs prior to membrane fusion in pleomorphic enveloped viruses or genome injection in tailed viruses, depending on the species (Prangishvili et al., 2017). Halorubrum Pleomorphic Virus-6 (HRPV-6), a non-lytic virus infecting *Halorubrum* sp. SS7-4, initiates infection by adsorbing to the host S-layer, which serves as the specific fusion receptor (Pietilä et al., 2012). Consequently, removal of the S-layer completely inhibits viral entry. While proteoliposomes reconstituted with the native host S-layer successfully fuse with HRPV-6, those containing proteoliposomes from related non-host haloarchaea do not (Bignon et al., 2022). This highly specific interaction is mediated by the VP4-like spike protein. Upon adsorption to the S-layer, the spike undergoes a conformational change, uncoiling into an elongated structure. Rather than disrupting the host matrix, this extension allows the spike to penetrate the porous, hexameric lattice of the S-layer to reach and fuse with the membrane (El Omari et al., 2019). Metagenomic analysis of *Pleolipoviridae* reveals highly diverse spike proteins, with phylogenetic relationships corresponding to host taxonomy. Consequently, pleolipoviruses exhibit very narrow host ranges, suggesting co-evolution of the S-layer and the viral S-layer binding protein (Alarcón-Schumacher et al., 2023).

Haloferax tailed virus 1 (HFTV1) is a lytic virus that infects *Haloferax gibbonsii* LR2-5 (Mizuno et al., 2019). Unlike other haloarchaeal viruses, which exhibit adsorption rate constants between 10^−10^ and 10^−13^ mL min^−1^, HFTV1 displays a rapid adsorption rate constant of 1.8 × 10^−9^ mL min^−1^ by binding to the S-layer. The host encodes two SLP genes, HfgLR_RS11205 and HfgLR_RS04600. Mutants resistant to HFTV1 possess mutations in HfgLR_RS11205, including complete deletions. These findings indicate that HfgLR_RS04600 alone is sufficient for S-layer function and that HfgLR_RS11205 likely serves as the primary HFTV1 receptor (Mizuno et al., 2019). The virus binds to the cell surface via both its head and tail. Its rapid binding may involve a two-stage mechanism: reversible head binding followed by irreversible baseplate binding (Schwarzer et al., 2023). This two-stage process may enhance binding efficiency, allowing the virus to rapidly secure attachment before committing to irreversible entry. The virus head features turret proteins with a putative deacetylase domain, which likely modifies S-layer N-glycans, facilitating entry by altering surface chemistry or exposing hidden receptors for baseplate binding (Schwarzer et al., 2023; Zhang et al., 2025). A glycosidase domain was also found in the crenarchaeal spindle-shaped virus SSV19 (Han et al., 2022). This enzyme is suggested to act not only in host recognition, but also in degrading glycans decorating the S-layer, potentially increasing accessibility or promoting local destabilization during entry (Zhang et al., 2025). Glycosidase activity has been confirmed for SSV19, which hydrolyses a glycan chain off the host surface (Yuan et al., 2025). Further studies examining how this activity affects S-layer structure could provide additional support for this model.

Another example is Halorubrum Tailed Virus DL1 (HRTV-DL1), a lytic virus infecting *Halorubrum lacusprofundi* ACAM34. Similar to HFTV1, both infection and adsorption are impaired in mutants with modified or deleted primary SLP, indicating it serves as the viral receptor. Adsorption is notably fast, with 75% of virions bound within 30 s of infection. The virion also features a potential sialidase domain, which may bind or degrade host surface (Mercier et al., 2023). However, disrupting host N-glycosylation does not affect the viral life cycle (Gebhard et al., 2023). HRTV-DL1 exhibits high host specificity, which may reflect precise adaption to the S-layer glycan composition of *H. lacusprofundi* ACAM34, limiting cross-strain infection (Mercier et al., 2023). The rapid adsorption kinetics of HRTV-DL1, similar to those of HFTV1, may reflect a conserved mechanism for efficient host recognition among S-layer-binding viruses.

The prevalence of S-layer binding among archaeal viruses remains uncertain due to the currently limited sample size. Notably, these viruses typically exhibit random surface-wide binding (Gehlert et al., 2022; Schwarzer et al., 2023). Similarly, other crenarchaeal viruses, such as the Sulfolobus monocaudavirus 1 (SMV1) from the *Bicaudaviridae* family, bind widely to abundant receptors at the surface of their hosts (Uldahl et al., 2016), suggesting that spindle-shaped viruses may frequently use the S-layer as a receptor. However, this is contrasted by observations that resistance to several *Fuselloviridae* is conferred by deletions in pilin genes (Rowland et al., 2020). Beyond serving as direct receptors (Quemin et al., 2013; Rowland et al., 2020; Wang et al., 2020; Hartman et al., 2019), pili may play additional roles in archaeal virus attachment. Recent evidence shows that archaeal type IV pili are capable of active retraction (Charles-Orszag et al., 2024). As some bacterial viruses exploit pilus retraction to approach the cell surface after initial attachment (Geiger and O’Toole, 2023; Chibeu et al., 2009; Meng et al., 2024; Thongchol et al., 2024; Bradley, 1974), archaeal viruses may similarly use retractile pili to facilitate subsequent engagement of secondary receptors, such as SLPs. Further studies are needed to test this possibility. Another shared trait among archaeal viruses is a narrow host range (Stedman et al., 2015; Alarcón-Schumacher et al., 2023; Mercier et al., 2023; Sensevdi et al., 2024). Glycosylation of the surface layer may also provide additional receptor diversity. Two archaeal viruses, the tailed virus φCh1 and the pleomorphic virus HRPV1, have been shown to bind to glycans on the archaeal surface. These glycans may be found on the S-layer or on other surface proteins (Klein et al., 2002; Kandiba et al., 2012).

Above, we reviewed examples of virus-S-layer interactions that reflect the structural diversity of archaeal cell surfaces (Alarcón-Schumacher et al., 2023; Schwarzer et al., 2023; Mercier et al., 2023; Tschitschko et al., 2018). We speculate that such diversification of S-layer sequences within a population serves as a first line of defense for archaeal hosts in general. Since the SLP provides an abundant receptor for viruses, generating structural variation is a simple yet effective counter-strategy.

Among *Haloferax* species, S-layer diversification occurs primarily through sequence recombination and is spread by vertical gene transfer in the genus (Shalev et al., 2018). On the other hand, the primary S-layer gene of *Hrbr. lacusprofundi* is located in a putative mobile genetic element adjacent to putative viral genes, while the primary S-layer gene of *Hrbr. marismortui* is plasmid-encoded (Calo et al., 2011; Tschitschko et al., 2015), suggesting involvement of horizontal gene transfer in S-layer diversification. However, comparison of *Haloferax* S-layer sequences indicates that they have rather been vertically inherited (Shalev et al., 2018). More data on S-layer sequences from other archaea are needed to clarify the relative contribution of horizontal and vertical gene transfer in S-layer diversification. The presence of a second SLP, as observed in several haloarchaeal species like *Hfx. gibbonsii* and *Hrbr. lacusprofundi*, further diversifies the archaeal cell surface (Mercier et al., 2023; Tittes et al., 2021). The secondary S-layer provides redundancy, allowing viable mutations in the major S-layer protein that functions as a viral receptor. In summary, multiple viruses from different families have recently been shown to bind archaeal S-layers (for an overview, see Table 1).

**Table 1:** Examples of S-layer glycoprotein-binding prokaryotic viruses.

Group	Virus name	Family (morphology)	Host organism	S-layer type	Host receptor	Viral anti-receptor	Reference
Archaeal viruses	SSV1	*Fuselloviridae* (spindle shape)	*Sulfolobus solfataricus*	Triangular lattice, heterodimer units	S-layer protein	Tail fiber protein	Zink et al. (2019)
	HRPV6	*Pleolipoviridae* (pleomorphic)	*Halorubrum* sp. SS7-4	Hexagonal lattice, monomer units	S-layer protein	VP4-like spike protein	Bignon et al. (2022)
	HFTV1	*Haloferuviridae* (head-tailed)	*Haloferax gibbonsii* LR2-5	Hexagonal lattice, monomer units	S-layer protein	Baseplate protein, potentially turret head proteins	Schwarzer et al. (2023)
	HRTV-DL1	*Flexireviridae* (head-tailed)	*Halorubrum lacusprofundi* ACAM34	Hexagonal lattice, monomer units	S-layer protein	Unknown, possesses baseplate proteins	Mercier et al. (2023)
	MetSV	*Tectiviridae* (spherical)	*Methanosarcina mazei* Gö1	Periodic, additional methanochondroitin	S-layer	Unknown	Gehlert et al. (2022)
	φCh1	*Vertoviridae* (head-tailed)	*Natrialba magadii*	Hexagonal lattice, monomer units	Mannose on surface, potentially S-layer	Tail fibre protein	Klein et al. (2012)
	HRPV-1	*Pleolipoviridae* (pleomorphic)	*Halorubrum* sp. PV6	Hexagonal lattice, monomer units	Legionaminic acid on the surface, potentially S-layer	VP4 spike protein	Kandiba et al. (2012)
	SSV19	*Fuselloviridae* (spindle)	*Sulfolobus* sp. E11-6	Triangular lattice, heterodimer units	Unknown, binds and hydrolyses S-layer glycans	Unknown, possesses VP4 spike protein	Han et al. (2022)
Bacterial viruses	ϕCD508	*Caudoviricetes,* formerly *Myoviridae* (myovirus)	*Clostridioides difficile*	Triangular prism lattice	S-layer protein	Baseplate RBP	Royer et al. (2023)
	ϕCD38-2	*Caudoviricetes,* formerly *Siphoviridae* (siphovirus)	*Clostridioides difficile*	Triangular prism lattice	S-layer protein	Baseplate RBP	Royer et al. (2023)
	ϕCD111	*Caudoviricetes,* formerly *Siphoviridae* (siphovirus)	*Clostridioides difficile*	Triangular prism lattice	S-layer protein	Baseplate RBP	Royer et al. (2023)
	ϕCD146	*Caudoviricetes,* formerly *Siphoviridae* (siphovirus)	*Clostridioides difficile*	Triangular prism lattice	S-layer protein	Baseplate RBP	Royer et al. (2023)
	ϕCD1801	*Caudoviricetes*, unclassified (siphovirus)	*Clostridioides difficile*	Triangular prism lattice	S-layer protein	Baseplate RBP	Whittle et al. (2022)
	ϕHN10	*Caudoviricetes*, unclassified (siphovirus)	*Clostridioides difficile*	Triangular prism lattice	S-layer protein	Baseplate RBP	Royer et al. (2023)
	AP50c	*Tectiviridae* (spherical)	*Bacillus anthracis*	Square lattice	S-layer protein	Spike protein p28	Forrest et al. (2023)
	Wip1	*Tectiviridae* (spherical)	*Bacillus anthracis*	Square lattice	S-layer protein	Spike protein p23	Forrest et al. (2023)
	Vp4	*Herelleviridae* (myovirus)	*Bacillus cereus* group	Square lattice	Sub-S-layer cell wall polysaccharides	Baseplate RBP	Nuytten et al. (2024)
	Deep-Blue	*Herelleviridae* (myovirus)	*Bacillus cereus* group	Square lattice	Sub-S-layer cell wall polysaccharides	Baseplate RBP	Nuytten et al. (2024)
	ϕCr30	*Caudoviricetes*, unclassified (myovirus)	*Caulobacter crescentus*	Hexagonal lattice	S-layer protein	Unknown	Edwards and Smit (1991)
	CNRZ 832-B1	*Caudoviricetes,* formerly *Siphoviridae* (siphovirus)	*Lactobacillus helveticus CNRZ 892*	Oblique or hexagonal lattice	S-layer protein SlpH (central domain loops of the protein monomer)	Unknown	Callegari et al. (1990)

## S-layer-mediated virus–host interactions in bacteria

S-layer binding has been commonly reported for bacterial viruses (see Table 1 for an overview). The S-layer of *Lactobacillus* spp. represents one of the best characterized phage adsorption structures among Gram-positive bacteria (Leprince and Mahillon, 2023; Villion and Moineau, 2009; Dieterle et al., 2014; Neviani et al., 1992). In the *Lactobacillus helveticus* phage CNRZ 832-B1 system, the virulent siphovirus recognizes the central variable region of the SLP SlpH (Callegari et al., 1990; Callegari et al., 1998; Quiberoni and Reinheimer, 1998). Studies of phage-resistant variants and sequence analyses of SlpH have implicated the central slpH domain in phage sensitivity while maintaining S-layer assembly. This indicates that the phage RBP specifically recognizes exposed structures within the S-layer lattice. Similar adsorption mechanism have been proposed for other *Lactobacillus helveticus* phages, emphasizing the role of the S-layer as a species and strain specific determinant of viral host range (Gatti et al., 2005).

In species such as *Lactobacillus brevis*, SLPs, including SlpA and SlpB, are extensively modified by O-linked and, in some cases, N-linked glycans (Hynönen and Palva, 2013). These exposed oligosaccharide motifs have been proposed as recognition targets for phage receptor binding proteins (RBPs) that contain lectin-like carbohydrate-binding modules (CBMs). Although direct structural evidence remains limited, genomic and biochemical studies support glycan mediated adsorption mechanisms in Lactobacillii and other Gram-positive bacteria. Modifications in the S-layer glycosylation pathway, such as mutations in glycosyltranserases or phase-variable expression of SLP genes, can confer resistance by masking or removing the carbohydrate epitopes recognized by phages. (Bertozzi Silva et al., 2016; Hynönen and Palva, 2013).

In the Gram-positive bacterial pathogen *Clostridioides difficile*, the S-layer functions as a principal receptor for both *Myoviridae* and *Siphoviridae*, serving as a common receptor despite considerable differences in the phage tail structures used for binding (Royer et al., 2023; Whittle et al., 2022). Bacteriophages targeting *C. difficile* utilize S-layer protein A (SlpA) and its associated secondary cell wall polymer (SCWP) for high affinity binding (Whittle et al., 2022). SlpA is the most abundant protein in the *C. difficile* S-layer and processed into two fragments: a high molecular fragment (HMF) that anchors in the cell wall, and a surface-exposed low molecular fragment (LMF) (Lanzoni-Mangutchi et al., 2022; Oatley et al., 2020). Certain phages, like the *C. difficile* myophage ϕCD508, possess exceptionally long tails (225 nm) that contract to generate sufficient energy to puncture both the S-layer and the thick peptidoglycan cell wall, without requiring auxiliary enzymatic domains to breach the wall (Wilson et al., 2025). In contrast, siphophages targeting *C. difficile* lack a contractile sheath and instead possess long, flexible, non-contractile tails. Rather than mechanically penetrating the cell envelope, these siphophages may rely on alternative tail-mediated or enzymatically assisted mechanisms to overcome the S-layer and cell wall following reversible binding (Leprince et al., 2026; Goulet et al., 2020; Heuler et al., 2021; Ramírez-Vargas et al., 2018).

The absence or alteration of SLP, such as deletion of the complete SlpA gene, renders the host completely resistant to the tested phages, demonstrating, demonstrating SlpA serves as primary and essential receptor (Royer et al., 2023). Studies on *C. difficile* highlight the complexity of SlpA as target receptor, with different phages exploiting diverse S-layer structures for entry (Royer et al., 2023; Royer et al., 2025). Understanding these interactions is vital to improving phage therapy against antibiotic-resistant bacteria like *C. difficile*. The high variability of S-layers among different strains explains the limited host range of many S-layer targeting phages, suggesting the need for specialized phage cocktails. (Dicks and Vermeulen, 2024; Dicks and Vermeulen, 2023)

The S-layer of *Bacillus* species determines virus–host interactions through its structural architecture and glycosylation, both of which can influence phage specificity (Plaut et al., 2014; Nuytten et al., 2024). In *Bacillus anthracis*, the S-layer consists primarily of two mutually exclusive proteins, Sap and EA1, with only one expressed at a time, depending on the bacterial growth phase, also called phase variation (Sogues et al., 2024; Missiakas and Schneewind, 2017). Sap predominates during exponential growth, whereas EA1 is expressed during the stationary phase (Mignot et al., 2002). Both proteins anchor noncovalently to pyruvylated secondary cell wall polysaccharides (SCWPs) via SLH domains, a process that depends on the enzyme CsaB (Missiakas and Schneewind, 2017; Boutonnet et al., 2022).

Several *Tectiviridae* phages utilize the S-layer as a receptor. The phage AP50c employs the trimeric RBP P28, together with the chaperone P29, to bind Sap monomers. As a result, AP50c can infect only hosts in the exponential phase, when Sap is present (Forrest et al., 2023; Bishop-Lilly et al., 2012). Deletion of *sap* in *B. anthacis* confers resistance to AP50c (Forrest et al., 2023). The closely related phage Wip1 uses P23, with P24, for Sap recognition, indicating a conserved mechanism among tectiviruses. Like AP50c, Wip1 is restricted to infecting Sap-expressing cells. Both phages lack long tail fibers and instead rely on compact, surface-exposed proteins for adsorption (Forrest et al., 2023). The temperate prophage λBa03 (Siphoviridae-like) encodes an RBP that requires an intact S-layer structure for binding, demonstrating that both lytic and temperate phages target this interface (Forrest et al., 2023). Cross-reactivity within the *Bacillus cereus sensu lato* group indicates evolutionary conservation of Sap receptor features. Sap homologues from *B. cereus* or *B. thuringiensis* can restore AP50c/Wip1 susceptibility in *B. anthracis Δsap* mutants, suggesting the presence of conserved S-layer binding motifs (Plaut et al., 2014).

In contrast, *Helleviridae* phages Vp4 and Deep-Blue appear to use multiple putative carbohydrate-binding modules (CBMs) within their baseplates to recognize polysaccharide receptors of the *Bacillus cereus* group, likely including secondary cell wall polysaccharides (SCWPs) associated with the cell envelope beneath the S-layer. Their putative baseplate hub proteins (Vp4 Gp110 and Deep-Blue Gp 189) exhibit ectolysin activity, locally degrading peptidoglycan to penetrate the S-layer and facilitate genome delivery (Nuytten et al., 2024).

Under phage pressure, *B. anthracis* can develop resistance by disrupting S-layer anchoring or presentation. Mutations in *csaB* prevent SCWP pyruvylation, resulting in detachment of Sap or EA1 and loss of phage receptors. These mutants display a mucoid phenotype due to altered extracellular matrix secretion. Direct mutations or deletions in *sap* abolish Sap synthesis, while mutations in the sp0A phosphorelay downregulate *sap* expression, resulting in decreased Sap abundance and reduced phage binding (Plaut et al., 2014; Forrest et al., 2023).

In summary, the S-layer structure and glycosylation are central determinants of phage-host specificity in *Bacillus*. The mutual exclusivity of Sap and EA1 introduces a temporal aspect to phage susceptibility, forcing phages to specialize in one SLP, which limits their host range, or to evolve broader mechanisms to overcome phase-dependent resistance. Phages such as AP50c and Wip1 target Sap directly, whereas *Helleviridae* phages employ ectolysins to bypass the S-layer. Host resistance emerges through genetic or regulatory disruptions to S-layer assembly, highlighting the dynamic co-evolution of phage virulence and host defense.

The S-layer of the Gram-negative bacterium *Caulobacter crescentus*, composed of the protein RsaA, serves as an obligate primary receptor for specific bacteriophages, including the transducing myophage φCr30 (Bharat et al., 2017; Edwards and Smit, 1991; Ely et al., 2015). This virus–host interaction is strongly influenced by cell-envelope glycosylation (Ardissone et al., 2014). The N-terminal domain of RsaA must anchor directly to the lipopolysaccharide (LPS) O-antigen of the host to form a stable and properly orientated lattice (Herdman et al., 2024; Andriko et al., 2020). Host mutations that disrupt this glycan-protein linkage, abolish S-layer secretion, or alter the S-layer structure itself, making the bacteria completely resistant to φCr30 infection. In contrast, *C. crescentus* can mitigate this selective pressure by producing an exopolysaccharide capsule that physically masks the S-layer and shields it from viral docking (Ardissone et al., 2014; Herr et al., 2018). The *C. crescentus* system demonstrates how spatial organization of SLPs and surface glycans determines viral susceptibility and drives the evolutionary arms race between receptor accessibly and phage evasion. While φCr30 binds to the RSA-containing S-layer on the cell surface, other *Caulobacter* phages like φCb13 and φCbK depend on the rotation or contraction of filamentous cell appendages, specifically flagella and pili, to facilitate attachment and movement towards the cell wall (Guerrero-Ferreira et al., 2011; Gill et al., 2012; Montemayor et al., 2020).

## Conclusion

In many archaeal systems and some bacteria, the S-layer glycoprotein itself functions as the primary host receptor (Sivabalasarma et al., 2025; Bertozzi Silva et al., 2016). Glycosylation patterns further refine virus–host specificity. Mutations or horizontal gene transfer can enable precise recognition or inhibition of viral attachment (Gebhard et al., 2023; Simpson et al., 2015). Prokaryotic viruses that target S-layers have developed different strategies to recognize and overcome the host’s glycosylation shield. Numerous phages encode glycosylation-degrading enzymes, typically located in their tails, that degrade extracellular polysaccharides (Simpson et al., 2015; Fernandes and São-José, 2018). These enzymes are primarily depolymerases (such as hydrolases and lyases) that allow viruses to penetrate bacterial capsules, biofilms, and extracellular polymeric substances (EPS) to initiate infection (Fernandes and São-José, 2018). Additionally, structural features such as immunoglobulin-like (Ig-like) domains enhance host interactions. These domains are commonly found in *Caudoviricetes* structural proteins and are incorporated through programmed ribosomal frameshifts (Fraser et al., 2006). Ig-like domains mediate reversible binding of glycans through weak electrostatic and carbohydrate-specific interactions (Fraser et al., 2007). This binding is particularly relevant for S-layer targeting, as demonstrated in *Lactobacillus* species. Furthermore, Ig-like domains contribute to adherence to mucus, highlighting their broader ecological significance (Rothschild-Rodriguez et al., 2023; Apjok et al., 2026). Notably, both archaeal S-layer and archaeal virus capsids are often glycosylated, and the presence of viral glycosyltransferases suggests roles in virion stability and host recognition (Gebhard et al., 2023; Sensevdi et al., 2024; Markine-Goriaynoff et al., 2004).

This interplay drives a co-evolutionary arms-race in which prokaryotes evade infection by altering their S-layer composition, expression or glycosylation patterns, while viruses counter-adapt to these defenses by exchanging receptor binding protein (RBP) genes to adapt to new hosts (Sensevdi et al., 2024; Simpson et al., 2015; Labrie et al., 2010; Koskella et al., 2022). These dynamics underscore the intricate balance between viral predation and host defense within microbial ecosystems (Leprince and Mahillon, 2023; Hampton et al., 2020; Weinbauer, 2004; Poullain et al., 2008; Weinbauer and Rassoulzadegan, 2004).

Despite variations in tail architecture, tailed *Caudoviricetes* frequently use SLPs as receptors, emphasizing their conserved role in viral adsorption. Ionic strength may influence viral attachment by modulating electrostatic interactions between virions and the cell, although most evidence for this effect is derived from bacterial systems, with archaeal systems remaining understudied (Wahid et al., 2026; Schaldach et al., 2006). Glycosylation of S-layer and other surface structures can further influence phage tropism by altering receptor accessibility and recognition. In bacteria such as *Clostridium difficile*, S-layer variability is a major determinant of virus–host range, and similar patterns are anticipated in archaea, pending further investigation (Royer et al., 2025). Research into phages that recognize pathogenic bacteria through their S-layer and associated glycosylation patterns is highly important because these surface structures strongly influence host specificity, infection efficiency, immune evasion, and the evolution of phage resistance. Understanding these interactions has major implications for precision phage therapy, anti-virulence strategies, diagnostics, and glycoengineering-based biotechnology applications (Dicks and Vermeulen, 2024; Schuster and Sleytr, 2015; Messner et al., 2008).

Future research should focus on identifying additional archaeal host receptors in order to enhance our understanding of virus–host interactions. High-resolution techniques, such as cryo-electron tomography, could reveal genome delivery mechanisms and the function of S-layers in facilitating viral infection. Complementary techniques, including surface plasmon resonance (SPR) or atomic force microscopy (AFM) could further elucidate virus-S-layer binding at the molecular level. Additionally, fluorescent live-cell imaging may resolve adsorption kinetics in real time, including type IV pilus retraction dynamics and viral genome delivery.

The prokaryotic virus examples discussed in this mini-review demonstrate the critical role of SLPs and their glycosylation in initiating host attachment and infection. The diversification of SLPs and their associated glycans represents an adaptable and highly specific defense mechanism, particularly in haloarchaea, where constant viral pressure necessitates dynamic surface barriers to block attachment and prevent genetic material injection. Studying S-layer-virus interactions and glycosylation-driven susceptibility is essential not only for understanding prokaryotic virus ecology and co-evolution, but also for enabling the rational design of therapeutics and engineered phages with improved targeting, stability, and clinical efficacy.
